# Conducting global mental health research: lessons learned from Kenya

**DOI:** 10.1017/gmh.2021.7

**Published:** 2021-03-08

**Authors:** Akash R. Wasil, Tom L. Osborn, Katherine E. Venturo-Conerly, Christine Wasanga, John R. Weisz

**Affiliations:** 1 Department of Psychology, University of Pennsylvania, Philadelphia, PA, USA; 2 Shamiri Institute, Nairobi, Nairobi County, Kenya; 3 Department of Psychology, Harvard University, Cambridge, MA, USA; 4 Department of Psychology, Kenyatta University, Nairobi, Kenya

**Keywords:** Anxiety, depression, implementation science, multicultural collaboration, school-based mental health

## Abstract

Mental health disorders are prevalent among youth and adolescents in low- and middle-income countries, and access to evidence-based treatments is poor. Although there is a great need for high-quality research to serve young people in low- and middle-income countries, there is limited guidance available for researchers who wish to conduct such work. Here, we describe our process of conducting school-based youth mental health work in Kenya over the last several years. We focus on five key lessons we learned that could guide future global mental health work with youth: (a) reducing stigma with strengths-focused interventions, (b) expanding access by working in schools, (c) generating buy-in from local stakeholders, (d) adapting the intervention via multicultural collaboration, and (e) applying insights from low- and middle-income countries to serve young people in high-income countries. We conclude by discussing how these lessons, and those shared by other teams, can be applied to help reduce the treatment gap for young people around the world.

## Commentary

As mental health problems and disorders are prevalent in low- and middle-income countries (LMICs) and access to treatment is limited, developing accessible treatments for young people in these settings has been a priority in global mental health research. Many researchers, who are primarily from high-income countries (HICs), have responded by initiating treatment studies for youths in LMICs. While these efforts can be valuable, they are often challenging. In addition to all of the ‘usual’ considerations involved in conducting high-quality research, investigators must address cultural, economic, logistical, political, and ethical considerations in LMIC settings.

As a result, researchers from HICs often encounter challenges – such as designing and adapting interventions that are appropriate for the culture and context; deploying interventions in settings that differ from those familiar to HIC investigators; forming and collaborating with multicultural research teams; and implementing interventions and assessments in ways that are appropriate for the participants. Researchers seeking to navigate these challenges may find limited guidance in the published literature. Essential details of the research and implementation process are rarely reported in publications or made publicly available (Murray *et al*., 2011; Singla *et al*., 2017; Watts *et al*., 2020). This presents an unfortunate paradox: there is a need for increased cross-cultural work to serve youths in LMICs, yet there is little guidance for investigators who seek to address that need. To fill this gap, it may be useful for investigators to share their experiences developing, implementing, and testing mental health interventions for young people in LMICs.

In this commentary, our multicultural team (consisting of investigators from the United States and Kenya) reflects on 3 years of performing adolescent mental health research in Kenya. We reflect on some of the challenges we faced, strategies we used to address those challenges, and lessons we learned. Given space limitations, we focus on a subset of challenges and lessons learned that we believe may be most relevant to other investigators pursuing their own global mental health research and promotion efforts.

## Shamiri: a school-based intervention for depression and anxiety

Our perspective is shaped by our work on *Shamiri* (Swahili for ‘thrive’), an intervention for adolescent depression and anxiety in Kenya (see https://www.shamiri.institute for details). The intervention includes three modules: gratitude, growth mindset, and value affirmation. It was first designed and evaluated as a school-based intervention for youths with elevated depressive and anxiety symptoms that were delivered over a 4-week period by lay counselors (Osborn *et al*., 2020c). In that pilot randomized controlled trial, conducted in 2018, Shamiri reduced depression symptoms and anxiety symptoms relative to an active study skills control condition. Guided by these findings, we performed a large-scale replication of the 4-week Shamiri, in which we found effects on depression and anxiety evident at post-treatment and persisting at 7-month follow-up (see Osborn *et al*., 2020b, 2020c for details). We have also found promising effects on depression in an online single-session adaptation of *Shamiri* (Osborn *et al*., 2020a). Efforts are currently underway to scale-up *Shamiri* across Kenya (see Alonge *et al*., 2020 for a detailed description of challenges and opportunities in scaling up school-based programs).

## Lessons learned

### Circumventing stigma with strengths-focused interventions

It is well-established that stigma around mental health inhibits help-seeking in many LMICs. In Kenya, for example, mental health problems are commonly viewed as signs of personal weakness, and stigma is common among youths (Ndetei *et al*., 2015). Thus, one challenge for intervention developers involves circumventing stigma.

One strategy for reducing stigma involves avoiding or reducing explicit reference to psychopathology that arises from terms such as ‘depression,’ ‘anxiety,’ ‘therapy,’ or ‘mental illness.’ Guided by this reasoning, we designed an intervention framed around *character strengths* rather than psychopathology. We drew inspiration from interventions within the positive psychology movement that are construed as cultivating strengths, improving relationships, and promoting well-being (Seligman, 2019). We reasoned that interventions focused on locally valued character strengths would be well received, even in contexts where traditional mental health interventions might confer stigma. Thus, we reviewed the literature on empirically supported character strength interventions and discussed this literature with Kenyan collaborators. This process – in which we first generated a list of evidence-based options and then narrowed it down with community partners – helped us select intervention modules that were evidence-based and culturally acceptable.

Our effort was evidently successful; students provided highly positive ratings of *Shamiri*'s acceptability and helpfulness. Additionally, school officials were enthusiastic about bringing *Shamiri* to their students. We credit this response in part to our decision to focus the intervention on character strengths. This focus appeared to circumvent stigma and to stimulate enthusiasm.

### Expanding access by working in schools

In many LMICs, government funding for mental health care is extremely low, and the mental health infrastructure is limited (Patel *et al*., 2007). As a result, one challenge for investigators involves designing innovative ways to deliver mental healthcare that do not rely on the kind of mental healthcare infrastructure that might be found in HICs. One strategy for addressing this challenge involves disseminating interventions in schools; this approach offers several advantages that are especially relevant in LMICs. First, delivering interventions in schools increase their *scalability*, as school-based interventions have the potential to reach a large portion of Kenyan youth. Second, school-based interventions are often *cost-effective*: they can draw on existing resources (e.g. classrooms, worksheets, and computers) and reduce transportation costs (see Wasil *et al*., 2020a for a cost-effectiveness evaluation of a school-based intervention in Kenya). Third, schools are a *natural setting* for learning and practicing skills. Previous research has shown that adolescents are accustomed to learning in school, which can enhance their ability to understand and retain mental health lessons (Chodkiewicz and Boyle, 2017). Additionally, adolescents encounter many stressors and challenges in schools, which makes schools a natural setting for students to apply and practice skills they learn from mental health interventions (Chodkiewicz and Boyle, 2017).

### Generating buy-in from local stakeholders

Securing buy-in from local stakeholders, such as school officials, can be challenging. We employed several strategies to engage school officials. First, as mentioned previously, we centered *Shamiri* around character strengths that appealed to school officials. Second, the inclusion of the study skills control condition was especially helpful in receiving buy-in from school officials. Because we included an active control condition, school officials were enthusiastic about the fact that students in both conditions could learn useful skills. Additionally, they expressed an especially strong interest in programs that could improve students' grades. Thus, we believe that one way of generating buy-in among school officials may involve including interventions that have a chance to improve students' academic performance. Third, we worked with school officials to ensure that the timing of intervention sessions did not conflict with other school events. Finally, to avoid tokenizing the opinions of local stakeholders (Jumbam, 2020), we were willing to make changes to our study design and intervention content based on feedback from local stakeholders. As an example, we initially brainstormed a longer version of Shamiri that included additional weeks of content. Feedback from school officials helped us develop the brief 4-week version of Shamiri. Using these strategies, we worked with 10 schools over the last 2 years.

### Adapting the intervention via multicultural collaboration

In addition to working with multicultural experts from Kenyan institutions to adapt our intervention, we relied on the perspectives of recent high school graduates in Kenya. One was a core member of our research team, and we recruited others as lay counselors. We reasoned that Kenyan high school graduates would be familiar with the pressures of the Kenyan education system, common challenges that high school students face, and potential barriers that could arise when delivering our intervention.

Importantly, the lay counselors were not only meant to deliver intervention content – we included them as active members of our team, and we worked with them to adapt our intervention content. We explained that our intervention was based on research, yet most of this research had been conducted with western samples. Therefore, while there were some aspects of our intervention that we were unlikely to change, there were many aspects of the intervention delivery that could be tailored for delivery in Kenya. Our approach was consistent with the idea that interventions consist of core components (i.e. essential and indispensable parts of an intervention) and an adaptable periphery (i.e. elements that can change based on the context in which the intervention is implemented; see Damschroder *et al*., 2009). To receive feedback on the adaptable periphery of our intervention modules, we prompted lay counselors to think about how high school students currently conceptualized each character strength (i.e. gratitude, growth, and values) and to discuss any challenges that could arise when delivering the intervention.

Using this process, we discovered several ways to adapt the content for Kenyan students. For example, we replaced the term ‘values’ with ‘virtues’ as Kenyan students were more familiar with the latter. Additionally, in our module on growth, we initially emphasized the malleability of personality traits (Schleider and Weisz, 2018). However, we learned that the term ‘personality’ was not universally understood and did not have a clear Kiswahili translation. As a result, we replaced references to ‘personality’ with those of ‘character traits’ and ‘personal qualities.’ Each lay counselor also shared personal stories related to each character strength: they shared a growth story (a time when they struggled at something and then improved through effort), reflected on things they were grateful for in a high school, and listed virtues that they found most important. We incorporated these examples into the intervention content and encouraged lay counselors to share these examples with students. Finally, the lay counselors helped us anticipate potential challenges that might occur during intervention delivery and brainstorm strategies to address those challenges.

### Applying Insights from LMICs to HICs

Global mental health work does not only have implications for those in LMICs. Just as interventions developed in high-income settings can be adapted for LMICs, lessons learned in LMICs can be ‘reverse-engineered’ to support individuals in HICs (Singla and Hollon, 2020). Each of the strategies we discussed are principles that could also be applied to enhance the impact of interventions in HICs as well. As an example, our work in LMICs (Osborn *et al*., 2020a; Wasil *et al*., 2020b) has informed recent efforts to support university students in the United States during the COVID-19 crisis (Wasil *et al*., 2020c). To adapt our interventions for American students, we applied the lessons we learned: we framed our interventions around character strengths to reduce stigma, deployed the interventions in universities to maximize reach, generated buy-in from university stakeholders, and worked with American students to adapt the interventions. In our experience, these strategies were useful both in LMICs and HICs, illustrating the importance of reverse engineering (Singla and Hollon, 2020).

## Conclusion

The growing enthusiasm for global mental health work has great potential to reduce the treatment gap around the world. In recent years, the importance of global mental health research has been emphasized in numerous review papers, commentaries, and commissions (Patel *et al*., 2007; Fazel *et al*., 2014; Singla *et al*., 2017; Patel *et al*., 2018). We hope our reflections add to this conversation by offering practical guidance to global mental health researchers.

Ideally, future research in global mental health will empirically test the best ways to overcome common challenges, including how to (a) select and adapt intervention content optimally, (b) deploy interventions in naturalistic settings, (c) generate buy-in from local stakeholders, (d) form and maintain multicultural collaborations, and (e) reverse-engineer interventions. Until this evidence base is thoroughly established, it will be useful for global mental health researchers to share the common challenges they encounter and the strategies they employed to address them. Such literature highlighting ‘lessons learned’ in global mental health research could help investigators implement studies more efficiently. Moreover, these resources could be especially helpful to individuals who have limited access to global mental health resources and mentors (e.g. scholars from disadvantaged backgrounds, early-career researchers, scholars at institutions where global work is rarely conducted). Thus, in addition to benefiting the global mental health community generally, these resources could also make global mental health work more accessible.

In conclusion, we encourage global mental health researchers to share their materials, reflections, and lessons learned. In addition to sharing intervention protocols (Watts *et al*., 2020), we believe there is a great need to share information about methodological, logistical, and implementation-related concerns that occur when conducting global work. Sharing such information openly could improve the quality and accessibility of global mental health research, ultimately helping investigators reduce the massive gaps in mental health care for young people worldwide.
